# ROI constrained optimal online allocation in sponsored search

**DOI:** 10.1038/s41598-024-77506-3

**Published:** 2024-10-29

**Authors:** Wenjian Xu, Shui Liu, Zulong Chen, Ying Zhou, Liangyue Li, Yangyang Wang, Quan Lu, Yuyang Ji, Jian Wan

**Affiliations:** 1 School of Information and Electronic Engineering, Zhejiang University of Science and Technology, Hangzhou, 310023 China; 2 Alibaba Group, Beijing, 100102 China; 3 Zhejiang lab, Hangzhou, 311121 China; 4 Linyi Vocational University of Science and Technology, Linyi, 276000 China; 5 Mashang Consumer Finance Co., Ltd., Chongqin, 401121 China; 6 School of Computer Science and Technology, Hangzhou Dianzi University, Hangzhou, 310023 China; 7 Zhejiang Key Laboratory of Biomedical Intelligent Computing Technology, Hangzhou, 310023 China

**Keywords:** Sponsored Search, Advertising systems, Online allocation, Auction mechanism, Engineering, Mathematics and computing

## Abstract

Sponsored search plays a major role in the revenue contribution of e-commerce platforms. Advertising systems are designed to maximize platform revenue, but other goals also need to be considered, such as user experience, advertiser utility, and how to achieve the long-term revenue goal. A key component of a sponsored search system is online allocation, which makes real-time decisions to match users’ search requests with relevant ad campaigns to maximize platform revenue within constraints such as campaign budgets. Although much progress has been made, most of the research work on allocation problem has focused on satisfying guaranteed deals for display ads, and those challenges for allocation problems in sponsored search are not properly addressed. In this paper, we develop a framework to solve the large-scale sponsored search ad allocation problem, consisting of two main parts. One is an optimization problem solved offline by a parameter-server based architecture, and the other is an online strategy to alleviate the conflict with the auction mechanism during online service. Comprehensive offline evaluation on real production data and online A/B testing on real production system have been made. The experimental results demonstrate that through better allocating user queries to appropriate ads, the proposed model can significantly increase the platform’s revenue without sacrificing advertisers’ ROI.

## Introduction

Sponsored search has always been an important part of e-commerce platform revenue generation. When a user issues a search query, search engines return the user organic search results along with sponsored ads on the same page. In this advertising system, platforms are incentivized to show ads that best match a user’s interests with advertiser’s bidding keywords, since platforms typically only get paid when a user clicks on an ad.

**Fig. 1:**
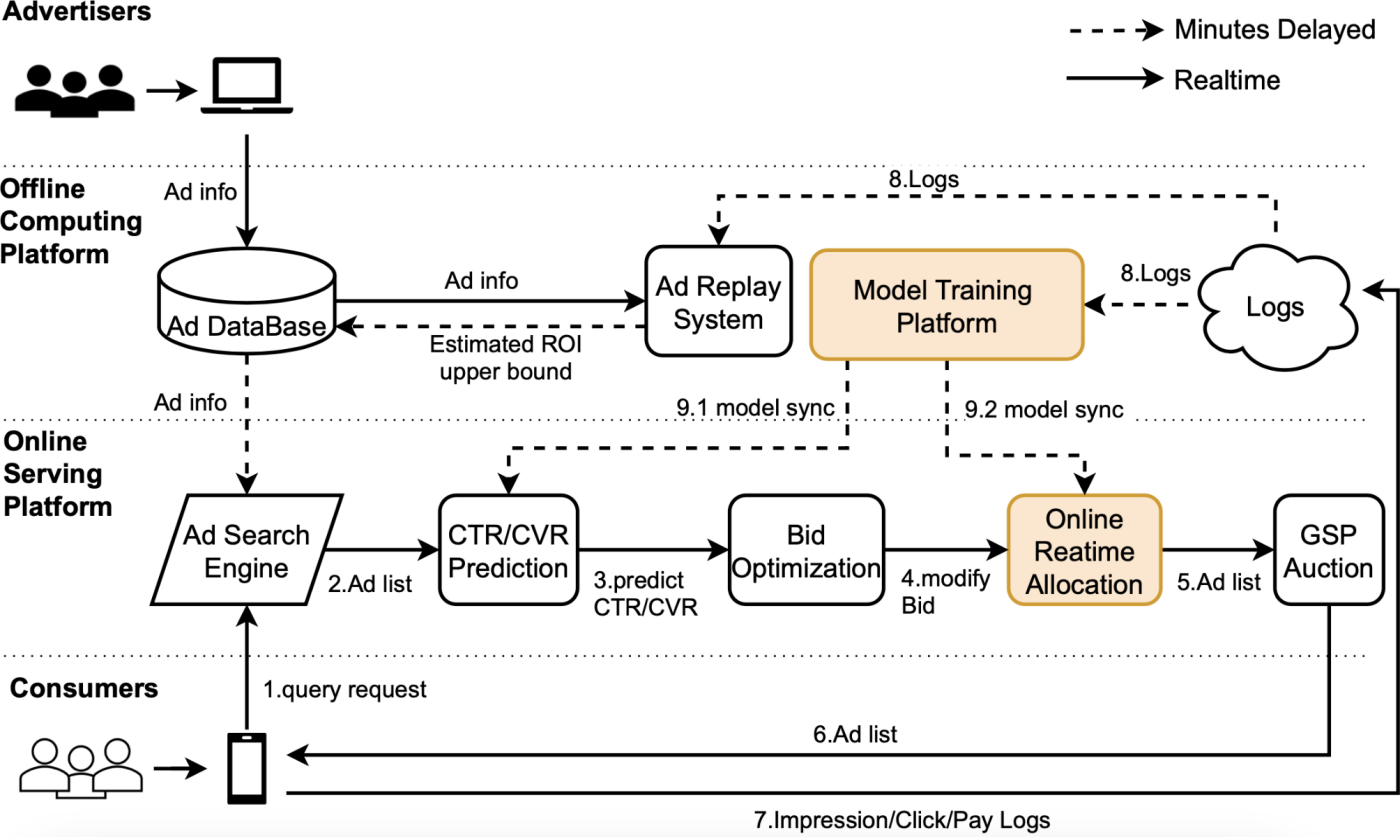
A typical sponsored search system.

Advertising systems are designed to maximize platform revenue by displaying relevant ads, also obligated to balance other key performance indicators (KPI), such as user experience, advertiser utility, and long-term revenue goal. A typical sponsored search system is shown in Fig. 1. Advertisers first place an order on the platform by setting target ad-words, target user group attributes, and desired bid and budget settings. In the online service stage, a candidate ads list is determined according to the match with the user’s search request (such as search query matching ad-words, etc.), and the subsequent prediction module will estimate the click-through rate (pCTR) and the conversion rate (pCVR) of each ad. After that, an optional bid optimization module modifies the bid price to maximize platform revenue and other KPIs. Next, the online allocation module, which is the focus of this paper, sets the eligibility of each ad to participate in the auction according to the allocation model trained offline. Finally, the ads participating in the auction are sorted in descending order according to their estimated cost per mile (eCPM = pCTR × Bid) value under the generalized second price mechanism (GSP), and the top ranked k ads are displayed to the user. If an ad in position $\:r$ is clicked, the advertiser will be charged with the bid price for ad in position $\:r+1$.

One of the key components of the sponsored search advertising system is the online allocation module, which maximizes platform revenue by better matching users’ search requests with relevant advertising campaigns in real time, while subject to some additional business constraints, such as campaign budget constraints. Most of the previous studies on online allocation module are in the area of display advertising with guaranteed deals^1–5^. Few studies have been done for sponsored search ads^6^. There are several reasons for this. First, sponsored search is performance-based and more performance-related constraints need to be considered, such like advertiser ROI constraints. Second, compared to the results of organic search, the results of sponsored search are less relevant to users’ search intent. Therefore, in terms of user experience, the platform may benefit from limiting the number of the displayed search ads. Third, there is a conflict between allocation models and GSP auction mechanism that allocation models rank ads by allocation probability while the GSP auction ranks ads by eCPM. To this end, the allocation result may have no effect on the advertising systems.

In this paper, we formulate the sponsored search ads allocation problem as a constrained optimization problem. In addition to typical campaign budget constraints, we also consider advertiser ROI constraints, where the lower bound satisfies the advertiser’s goals and the upper bound ensures the stability of the advertising ecosystem. Since displaying too many ads will impact users’ search experience^7,8^, in addition to platform revenue, we directly put maintaining a certain level of user experience as part of our target functionality.

Our new proposed model is called ROAM (ROI constrained Optimal Allocation Model). To solve it efficiently, a parallel optimization algorithm is developed based on the parameter server architecture, and generates a compact allocation plan for online serving. For the conflict between allocation model and GSP auction mechanism, an online strategy is designed. Comprehensive experiments have been conducted both offline and online on the real production data demonstrating that the proposed model can achieve significant improvements in both advertising platform’s revenue and advertiser’s ROI. The main contributions of our work are summarized as follows:

- We propose a new allocation model that simultaneously optimizes platform revenue and user experience, and handles advertiser ROI constraints, one of the most important business constraints.
- We develop a parallel optimization algorithm based on parameter server framework to solve ROAM efficiently, and design an online serving strategy that resolves the conflict between allocation model and GSP auction mechanism.
- In online and offline experiments, our method improves revenue significantly compared to previous methods without sacrificing ROI. After more than three months from the first launch in production environment, our method is still running stably and works efficiently.

## Related work

In this section, we conduct a brief survey on online allocation and sponsored search.

### Online allocation

In online advertising area, allocation algorithms are used mainly for two different purposes: one for optimizing campaign (locally) and one for optimizing platform (globally).

When optimizing campaign, allocation algorithms are used to control the rate at which individual campaign’s budget is spent. Most commonly, for each search query, an allocation algorithm calculates the probability of serving each ad, and then applies throttling based on those probabilities^9–11^.

When optimizing platform, allocation algorithm is typically formulated as a constrained optimization problem based on graph matching. Please refer to^12^ for a detail and comprehensive survey. Chervonenkis et al.^13^ find optimal ads allocation in sponsored search by relaxing the original integer programming to a continues optimization problem. Different approaches are proposed to deal with allocation problems with different objectives. For instance, Abrams et al.^14^ formulate the allocation problem as a Linear Programming (LP) problem and applies column-generation method to solve it. However, it has limited scalability and can only be applied to high frequency queries. Zhang et al.^15^ propose a consumption minimization model, in which the primary objective is to minimize the user traffic consumed to satisfy all advertisement contracts in online display advertising. Their method is based on finding the max flow solution for a bipartite graph matching problem. The authors in^6,16^ reduce the number of variables of LP problem using the a primal-dual approach and obtain optimal solutions through offline simulations with historical data to maximize total revenue and other key performance of the auction based advertising subject to budget constraints. High Water Mark (HWM)^1^ and SHALE^2^ are both allocation models proposed for guaranteed display ads. They try to minimize under-delivery penalty as well as the gap between allocation probability and supply-demand ratio through iterative offline optimization method. The authors in^3,4,17^ extend SHALE to address large scale allocation problem. In addition to guaranteed delivery, they also consider other types of real business needs, such as optimizing click-through rates, penalizing over-allocation, and meeting frequency requirements.

Inspired by previous work above, we design a scalable allocation model for sponsored search. It combines two goals. One of them is to maximize advertising revenue and the other is to limit user experience degradation caused by displaying ads. Regarding constraints, it includes not only campaign budget constraint but also campaign ROI constraint.

### Sponsored search

Researchers have focused on various aspects to enhance the effectiveness and efficiency of sponsored search advertising. Progress has been achieved in applying reinforcement learning algorithms to optimize advertising strategies, exploring the potential of transfer learning in different advertising scenarios, and improving personalized ad recommendations through user modeling. For instance, Lian et al.^18^ propose a novel model-free reinforcement learning approach to select the best K items from a pool of N candidates, provided by the upstream, in order to maximize the total system revenue. One line of work^19,20^ enhances ad retrieval and ranking using advanced neural network-based methods, and another line of work^21–23^ shares a common focus on leveraging graph-based approaches to improve the understanding of user intentions and enhance relevance modeling. Recently, Tian et al.^24^ argue that the diverse and personalized preferences of users and advertisers are ignored existing relevance models. To address this issue, the authors incorporate the portraits of users and advertisers into the relevance models.

Researchers have developed joint optimization and multi-objective optimization approaches to balance the interests of advertisers and users, considering factors like cost, revenue, and user satisfaction. Liu et al.^25^ propose CIA, an impression-level bidding optimization framework that satisfies various e-commerce advertiser demands, unifying the benefits of all parties in the marketing ecosystem. Yang et al.^26^ introduce AiAds that enhances campaign performance, user experience, and advertising platform revenue with a bidding language and allocation mechanism, thereby overcoming limitations of keyword targeting. Zhao et al.^27^ suggest manipulating ad titles by adding selling point keywords (SPs) to attract query users, benefiting merchants, the platform, and enhancing user experience. Guan et al.^28^ present MACG, a multi-objective cooperative bid optimization framework that maximizes overall profit and prevents collusion with a platform revenue constraint. Recently, Ou et al.^29^ review the latest bidding strategies for online advertising platform, and it helps researchers to gain insights of the challenges and future prospects of bid optimization.

The research community has also emphasized privacy protection and fairness in sponsored search by investigating methods to safeguard user privacy and ensuring fair and sustainable advertising systems. Li^30^ introduces a dynamic reserve price design framework to provides long-term incentives for advertisers to be honest about their valuations while minimizing the impact on user experience. These advancements offer new perspectives and methodologies for the ongoing development and improvement of sponsored search, fostering better user experiences and driving positive impacts in the advertising industry.

## The proposed methodology

**Fig. 2:**
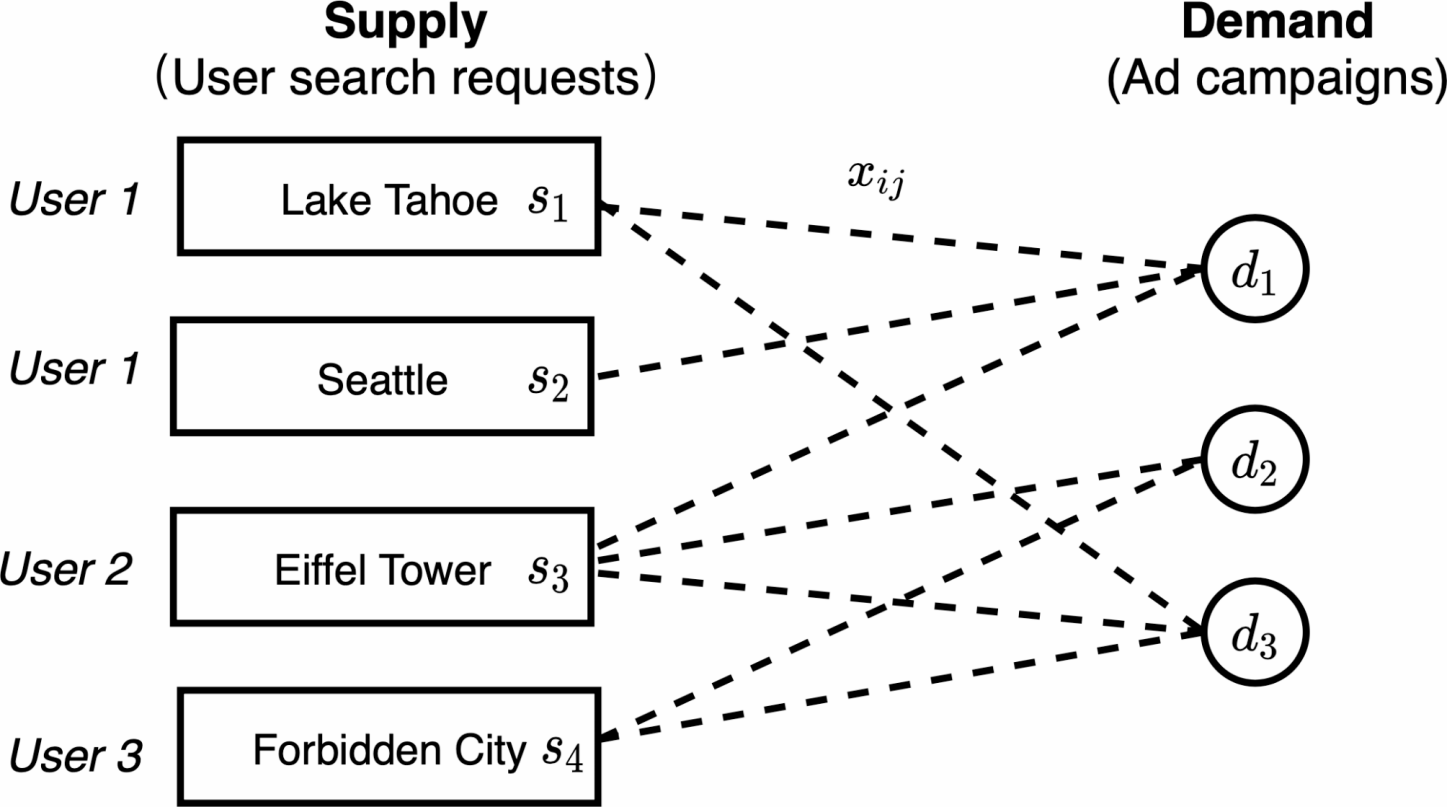
Bipartite graph of supply and demand in sponsored search ad system.

The ad allocation problem is usually modeled as a bipartite graph matching problem with some constraints^2,17^ as illustrated in Fig. 2. Let $\:G=(I\cup\:J,E)$ be a bipartite graph, where there are two types of nodes, i.e., the supply nodes $\:i\in\:I$ that represent user’s search requests belonging to a certain query type and the demand modes $\:j\in\:J$ that represent the ad campaigns. One supply node is connected with a demand node if the user’s search query matches the campaign’s target ad words and the user is in the campaign’s target user group. Each demand node $\:j$ has a budget $\:{d}_{j}$ set by the advertisers and each supply node $\:i$ has a weight $\:{s}_{i}$ indicating the number of user requests belonging to a certain query type.

### Basic allocation problem formulation

The task of the allocation problem is to find the optimal allocation probability $\:{x}_{ij}$, i.e., the fraction of the supply node $\:i$ allocated to demand node $\:j$, that (1) maximizes some objective functions, and (2) satisfies some constraints. In this paper, we want to maximize the platform revenue with the minimum ads impressions to minimize the disruption to the organic search results. If one impression from supply node $\:i$ is allocated to ad $\:j$ on the demand side, the platform would charge the advertiser $\:{c}_{ij}$ and the advertiser would gain revenue from potential sales $\:{g}_{ij}$, where $\:{c}_{ij}={pCTR}_{ij}\times\:{pCPC}_{ij}$ and $\:{g}_{ij}={pCTR}_{ij}\times\:{pCVR}_{ij}\times\:{price}_{j}$. Note that, since $\:{CPC}_{ij}$(Cost Per Click) is not available prior to GSP auction, we use average historical $\:{CPC}_{j}$ of ad $\:j$ as $\:{pCPC}_{ij}$ instead. The click-through rate $\:{pCTR}_{ij}$ and the conversion rate $\:{pCVR}_{ij}$ are generated from a separate predict model, $\:{pCPC}_{ij}$ is the cost per click charged to advertisers and $\:{price}_{j}$ is the price of the product/service sold by the advertiser. Considering these, the optimal allocation problem in sponsored search can be formally defined as:

1 $$\:\underset{x}{\text{m}\text{ax}}\sum\:_{i\epsilon{\Gamma}\left(j\right),j}{s}_{i}{x}_{ij}{c}_{ij}-\lambda\:\sum\:_{i\epsilon{\Gamma}\left(j\right),j}{s}_{i}{x}_{ij}^{2}$$

$$s.t. \quad \:\sum\:_{i\epsilon{\Gamma}\left(j\right),j}{s}_{i}{x}_{ij}{c}_{ij}\le\:{d}_{j},\:\forall\:j \quad \text{(budget constraint, 1a)}$$

$$\:\sum\:_{j\in{\Gamma}\left(j\right),j}{x}_{ij}\le\:1,\forall\:i \quad \text{(supply constraint, 1b)}$$

$$\:{u}_{j}\ge\:\frac{\sum\:_{i\in\:{\Gamma}\left(j\right)}{s}_{i}{x}_{ij}{g}_{ij}}{\sum\:_{i\in\:{\Gamma}\left(j\right)}{s}_{i}{x}_{ij}{c}_{ij}}\ge\:{l}_{j},\forall\:j \quad \text{(ROI constraint, 1c)}$$

$$\:{x}_{ij}\ge\:0,\forall\:i,j \quad \text{(non-negativity constraint, 1d)}$$

where nodes in $\:{\Gamma\:}\left(j\right)$ are the neighbors of node $\:j$ in the bipartite graph. The objective is to maximize the platform revenue $\:\left({s}_{i}{x}_{ij}{c}_{ij}\right)$ while minimizing the impressions allocated $\:\left({s}_{i}{x}_{ij}^{2}\right)$, i.e. minimizing the negative impacts to user experience. The hyper-parameter $\:\lambda\:$ balances the two aspects.

**Constraints**. *Budget constraint (Eq. 1a)*: Each ad campaign has a budget $\:{d}_{j}$ set by the advertiser and the total cost of a campaign should not exceed its budget. *Supply constraint (Eq. 1b)*: this should be obvious since the total allocation from a supply node $\:i$ should not exceed its capacity. *ROI constraint (Eq. 1c)*: the return over investment is defined as the ratio of the sales from the ads placed $\:\left(\sum\:_{i\in\:{\Gamma\:}\left(j\right)}{s}_{i}{x}_{ij}{g}_{ij}\right)$ over the cost charged to the campaign $\:\left({\sum\:}_{i\in\:{\Gamma\:}\left(j\right)}{s}_{i}{x}_{ij}{c}_{ij}\right)$. Usually, the advertisers set up a minimum ROI $\:{l}_{j}$ that we are obliged to guarantee, which implies the least sales revenue that the advertise can achieve with the budget invested. Furthermore, we set an upper limit on ROI for each campaign based on the historical data through a replay mechanism. On one hand, the upper limit of ROI is to ensure the stability of the ROI of advertisers, to prevent the ROI of advertisers from being very high when there is no competition, but falling a lot when the competition is fierce. The stability of ROI allows advertisers to make better marketing schedules in advance. On the other hand, the upper limit of ROI is set to reserve some high-quality traffic to improve the performance of ads with extremely low ROI, so as to avoid such advertisers churn on the platform.

### Optimization algorithm

The allocation problem in Eq. (1) is an optimization problem with convex objective and linear constraints. We can obtain its optimal solution by solving its dual problem through the KKT condition. More specifically, the corresponding Lagrangian function is

2 $$\begin{aligned} \:L\left(x,\alpha\:,\beta\:,\varphi\:,\eta\:,\zeta\:\right)=\sum_{i\epsilon{\Gamma}\left(j\right),j}{s}_{i}{x}_{ij}{c}_{ij}-\lambda\:\sum_{i\epsilon{\Gamma}\left(j\right),j}{s}_{i}{x}_{ij}^{2}+\sum_{j}{\alpha}_{j}\left(\sum_{i\epsilon{\Gamma}\left(j\right),j}{s}_{i}{x}_{ij}{c}_{ij}-{d}_{j}\right) \\ \:+\sum_{i}{\beta}_{i}\left({s}_{i}\sum_{j\in\:{\Gamma}\left(\text{i}\right)}{x}_{ij}-{s}_{i}\right)+\sum_{j}{\eta}_{j}\left({l}_{j}\sum_{i\in\:{\Gamma}\left(j\right)}{s}_{i}{x}_{ij}{c}_{ij}-\sum_{i\epsilon{\Gamma}\left(j\right)}{s}_{i}{x}_{ij}{g}_{ij}\right) \\ \:+\sum_{j}{\zeta}_{j}(\sum_{i\in\:{\Gamma}\left(\text{j}\right)}{s}_{i}{x}_{ij}{g}_{ij}-{u}_{j}\sum_{i\in\:{\Gamma\:}\left(\text{j}\right)}{s}_{i}{x}_{ij}{c}_{ij})-\sum_{i\epsilon{\Gamma}\left(j\right),j}{\varphi\:}_{ij}{x}_{ij}\:\end{aligned}$$

From the KKT stationarity condition of $\:\frac{\partial\:L}{\partial\:{x}_{ij}}=0$ and the complementary slackness for $\:{\varnothing\:}_{ij}$, i.e., $\:{\varnothing\:}_{ij}=0$ unless $\:{x}_{ij}=0$, we have:

3 $$\:{x}_{ij}=\text{m}\text{a}\text{x}\{0,\lambda\:{c}_{ij}-{\alpha\:}_{j}{c}_{ij}-{\beta\:}_{i}-{\eta\:}_{j}\left({l}_{j}{c}_{ij}-{g}_{ij}\right)-{\zeta\:}_{j}\left({g}_{ij}-{u}_{j}{c}_{ij}\right)\}$$

which is a function of $\:{\alpha\:}_{j},{\eta\:}_{j}$ and $\:{\zeta\:}_{j}$, denoted by $\:{x}_{ij}=f({\alpha\:}_{j},{\beta\:}_{i},{\eta\:}_{j},{\zeta\:}_{j})$. The dual variables $\:\alpha\:,\eta\:$ and $\:\zeta\:$ can be solved iteratively by coordinate descend or gradient descent algorithm until the objective function converges. The gradients of $\:\alpha\:,\eta\:$ and $\:\zeta\:$ are calculated as:

4 $$\:\frac{\partial\:L}{\partial\:{\alpha\:}_{j}}=\sum\:_{i\epsilon{\Gamma}\left(j\right),j}{s}_{i}{x}_{ij}{c}_{ij}-{d}_{j}$$

5 $$\:\frac{\partial\:L}{\partial\:{\eta\:}_{j}}={l}_{j}\sum\:_{i\epsilon{\Gamma}\left(j\right)}{s}_{i}{x}_{ij}{c}_{ij}-\sum\:_{i\epsilon{\Gamma}\left(j\right)}{s}_{i}{x}_{ij}{g}_{ij}$$

6 $$\:\frac{\partial\:L}{\partial\:{\zeta\:}_{j}}=\sum\:_{i\epsilon{\Gamma}\left(j\right)}{s}_{i}{x}_{ij}{g}_{ij}-{u}_{j}\sum\:_{i\epsilon{\Gamma}\left(j\right)}{s}_{i}{x}_{ij}{c}_{ij}$$

Since the number of supply node is usually very large, we propose an efficient parallel algorithm to solve the allocation model using a Parameter-Server architecture, detailed in Algorithm 1. At first iteration, we calculate $\:{\beta\:}_{i}$ with zero as initial values of $\:{\alpha\:}_{j},{\eta\:}_{j},{\zeta\:}_{j}$. After that, in each iteration on worker side, $\:{beta}_{i}$ is calculated with equation $\:\sum\:f\left({\alpha\:}_{j},{\beta\:}_{i},{\eta\:}_{j},{\zeta\:}_{j}\right)=1$, and $\:{x}_{ij}$ can be obtained with Eq. (3), then on server side $\:{s}_{i}{x}_{ij}{c}_{ij}$, $\:{s}_{i}{x}_{ij}{g}_{ij}$ are gathered to update $\:{\alpha\:}_{j},{\eta\:}_{j},{\zeta\:}_{j}$ with Eq. (4) to Eq. (6). As shown in Algorithm 1, in the worker side, the time complexity is $\:O\left(\right|I\left|*\stackrel{\sim}{{\Gamma\:}}\right)$, where $\:\stackrel{\sim}{{\Gamma\:}}$ is the average number of neighbors for each node in the bipartite graph. In the server side, the time complexity is $\:O\left(\right|J\left|\right)$.


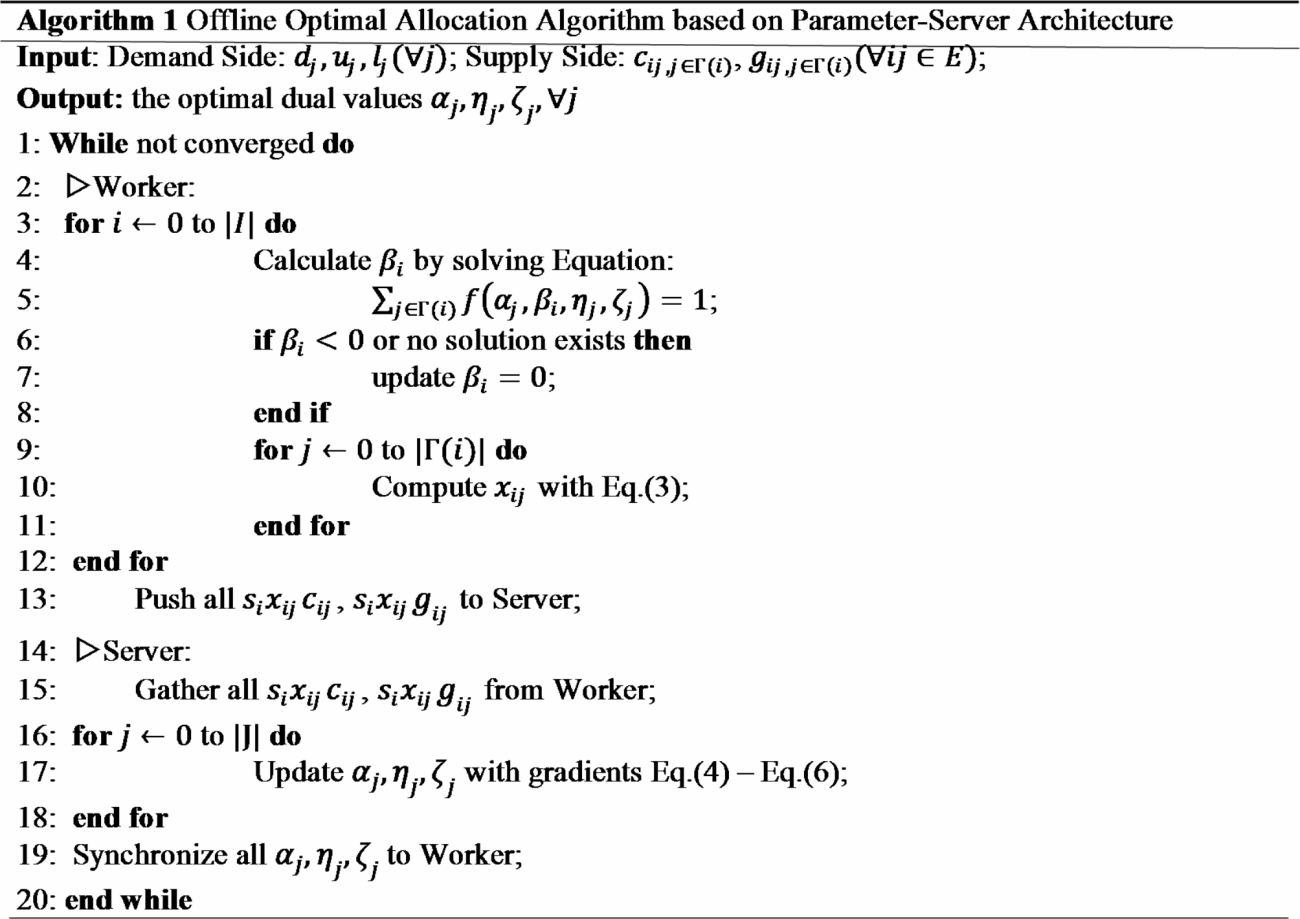


### Online serving

In the multiple representation learning framework, if the sub-networks are employed directly as in MMOE and PLE, the sub-networks would learn similar features that are very closely distributed in the representation space since they are fed with the same CTR labels. The worst case is that all the sub-networks collapse to the same space, which is harmful for dealing with sparse and long-tail data.

As shown in Algorithm 2, during the online service process, firstly, a set of candidate ads that best match user’s search request is selected. Then $\:{\beta\:}_{i}$ is solved for each request $\:i$ with equation $\:\sum\:_{j\in\:{\Gamma\:}\left(i\right)}f\left({\alpha\:}_{j},{\beta\:}_{i},{\eta\:}_{j},{\zeta\:}_{j}\right)=1$ ($\:{\alpha\:}_{j},{\eta\:}_{j},{\zeta\:}_{j}$ are solved in offline stage). After that, for each ad $\:j$ in this list, allocation probability $\:{x}_{ij}$ can be computed by Eq. (3). Note that ad $\:{j}^{*}$ with highest $\:{x}_{ij}$ value among them may not be the winning ads in later auction process, because it may not have the highest eCPM value. However, to maximize platform’s revenue, which is proportional to the winning ad’s second price, we want ad $\:{j}^{*}$ to be always the winning ads in auction. To solve this conflict between allocation and GSP auction, our allocation algorithm put all ads whose eCPM is lower than the eCPM of ad $\:{j}^{*}$, to be reserved to participate in the auction, as described in line 8 to line 19 of Algorithm 2.


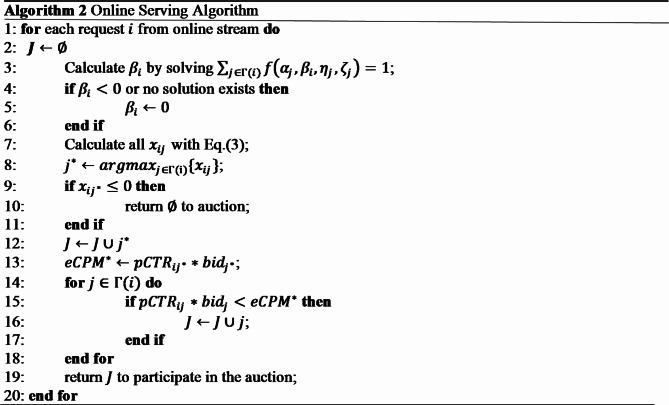


## Experiments

In this section, we first describe some offline experimental results on logged production datasets. It includes comparison and analysis of the convergence properties and key performance of different models on these datatsets and a sensitivity analysis of the hyperparameter values in our model ROAM. We also run an online A/B test to test our allocation model’s performance at runtime. We use a throttling-based algorithm as a baseline model. The results show that our model outperforms the baseline model in terms of both the platform’s RPM and the advertiser’s ROI.

### Experimental setting

#### Data sets and evaluation metrics

We use a dataset with 1.2 million requests (supply node), 622 ad campaigns (demand nodes), and more than 4.8 million edges in the allocation bipartite graph created from sampling of logs dumped from production system. Note that the true click and conversion probability of each ad is still unknown even for offline model evaluation. We use model estimated CTR and CVR to represent the true values instead.

For the offline evaluation, we consider the following metrics:

- **Budget Consumption Rate (BCR)** is the ratio of total estimated revenue under allocation to total budget, and how close the offline evaluation result achieved by the methods to the upper bound can be observed by this metric. Budget consumption rate of offline evaluation is calculated as:

$$\:BCR=\frac{\sum\:_{i\in\:{\Gamma}\left(j\right),j}{x}_{ij}{c}_{ij}}{\sum\:_{j}{d}_{j}}$$

- **Revenue Per Mile (RPM)** is a metric that represents how much money platform can earn per 1000 impressions. In our offline test context, the RPM evaluation is calculated as:

$$\:RPM=1000*\frac{\sum\:_{i\in\:{\Gamma}\left(j\right),j}{x}_{ij}{c}_{ij}}{\sum\:_{i\in\:{\Gamma}\left(j\right),j}{s}_{i}{x}_{ij}}$$

- **Return on Investment (ROI)** tries to directly measure the amount of return on advertiser’s displayed ads, relative to their advertising cost. In out offline test scenario, advertiser’s ROI is calculated as:

$$\:ROI=\frac{\sum\:_{i\in\:{\Gamma}\left(j\right),j}{x}_{ij}{g}_{ij}}{\sum\:_{i\in\:{\Gamma}\left(j\right),j}{x}_{ij}{c}_{ij}}$$

#### Benchmark methods

For offline allocation algorithm comparison, we compare the performance of our proposed method with four commonly used ones plus a variant version of ROAM, which has excluded the ROI constraint. To guarantee the fairness, we use the same objective function as described in our problem definition for all the methods.

- *HWM* is proposed by^1^. It first sorts all contracts in decreasing order of demand-supply ratio, then allocates each contract an equal portion from each eligible supply.
- *SHALE* is proposed by^2^, and it is modeled to minimize penalty and maximize representativeness, which is a measure of how close the allocation result is to demand-supply ratio.
- *AUAF* is proposed by^4^. It is derived from SHALE with the main objective of maximizing the contract delivery rate. It also aims to maximize click through rate and avoid over-allocation.
- *ODBC* is proposed by^6^, which formulates the allocation problem as a single objective linear programming problem to maximize revenue with the constraints of CTR and CVR in campaign level. One simplifying assumption made in this algorithm is that the distribution from which impressions are drawn is stationary, which means given sufficient historical data, optimal priori values of the dual variables can be learned by solving the dual offline.
- *OAM* (ROAM without ROI constraint) is to demonstrate the effectiveness of ROI constraint in our proposed model. Specifically, the ROI constraint (i.e., Inequation 1c in problem definition) is removed from ROAM.

For models like HWM, SHALE and AUAF, demand-supply ratio is a key input to the representativeness objective. We have to first convert our ad campaign budgets demand to impressions demand, and calculate the demand-supply ratio, then we can apply these models to our allocation problem.

### Offline allocation evaluation

**Table 1:** Offline evaluation results (GMV is short for gross merchandise volume, GMV=$\:\sum\:_{i,j}{s}_{i}{x}_{ij}{g}_{ij}$).

Methods	Revenue	RPM	BCR	GMV	ROI
HWM	21259.32	53.31	13.33%	70015.95	3.2934
SHALE	23291.12	58.40	14.60%	83173.81	3.5692
AUAF	25901.58	64.95	16.24%	94950.61	*3.6658*
ODBC	**49571.92**	**124.30**	**31.09%**	**173054.56**	3.4910
OAM	*40278.77*	*101.00*	*25.26%*	135346.46	3.3597
ROAM	39444.78	98.91	24.73%	*148571.29*	**3.7601**

**Comparison Results.** We show the comparison results of different methods on five key performance indicators in Table 1. ODBC’s performance on all the indicators except ROI can be regarded as the upper bounds for other methods since it does not consider the ROI constraint. The proposed ROAM achieves the highest ROI while maintaining competitive Revenue, RPM, BCR and GMV.

**Fig. 3:**
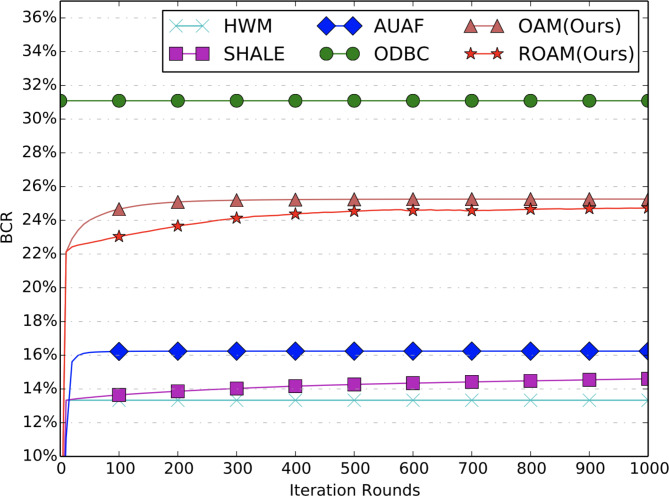
BCR curves of offline allocation.

**Fig. 4:**
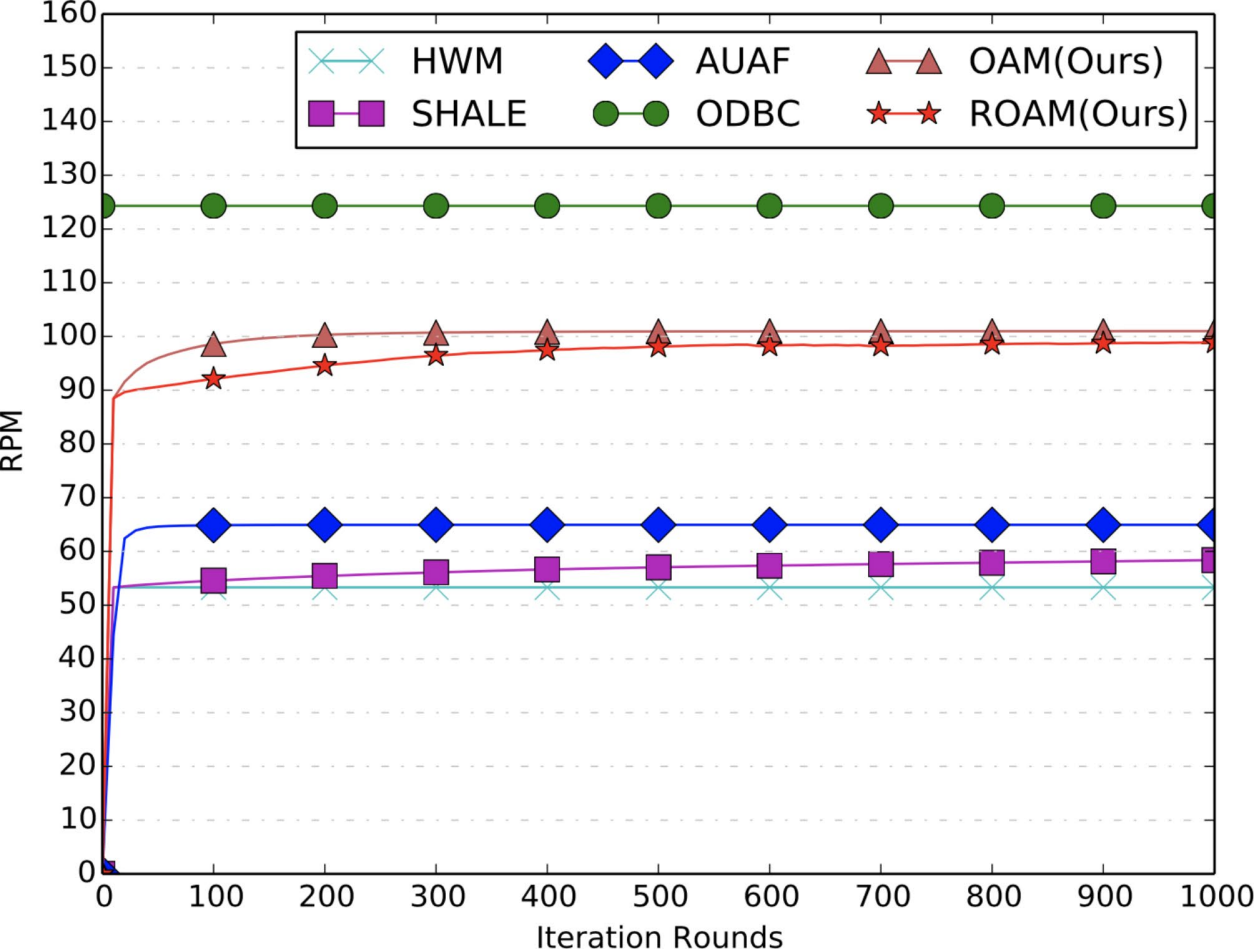
RPM curves of offline allocation.

**Fig. 5:**
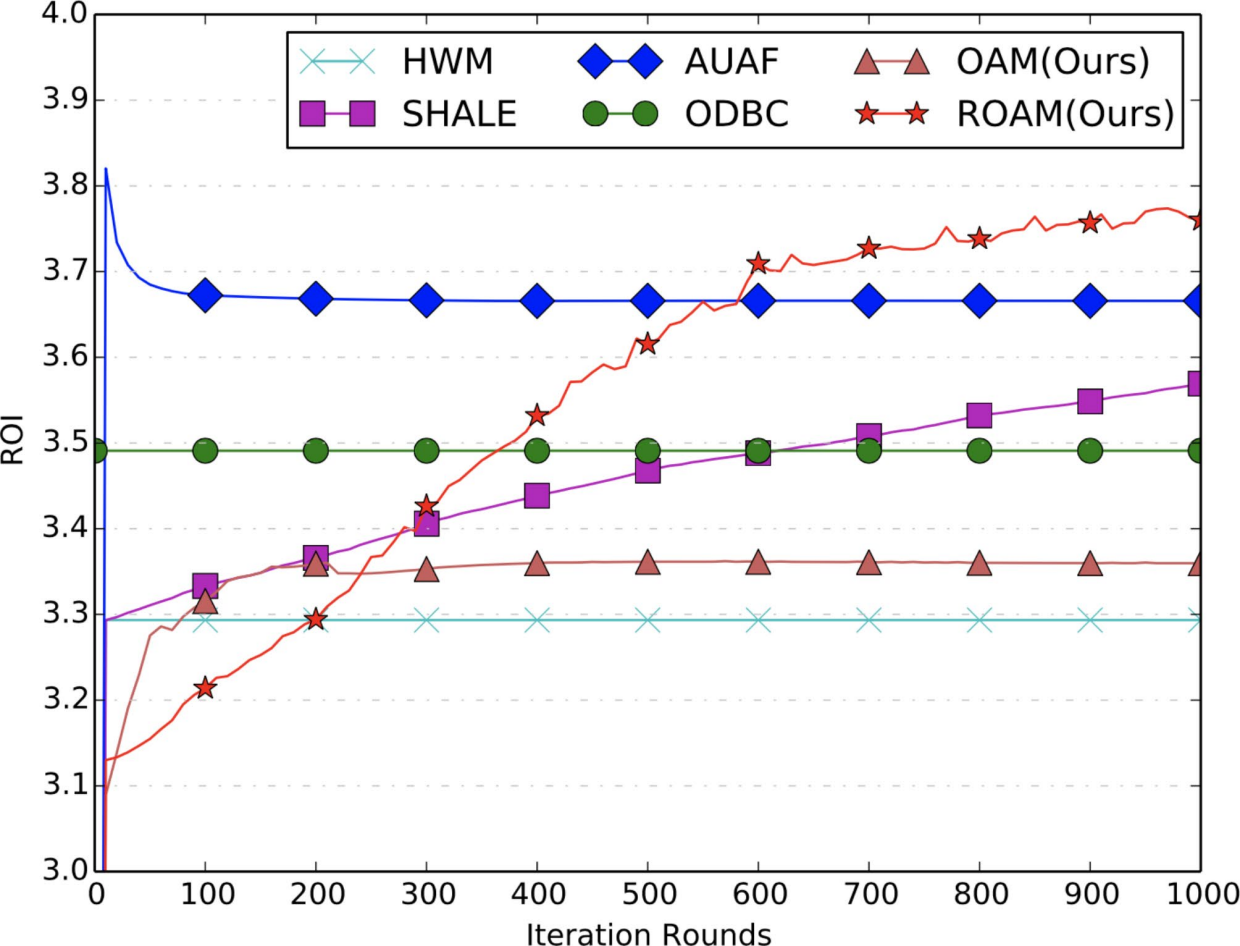
ROI curves of offline allocation.

**Convergence Analysis.** From Figs. 3, 4 and 5, we can see that all the comparison methods converge within 200 iterations on both BCR and RPM metrics. Note that, results for ODBC are shown as a horizontal line since it is solved by an open-sourced LP solver without the iteration procedures. HWM only runs through the data for only one round, hence it is also shown as a horizontal line. In fact, its performance equals that of SHALE after one iteration. The convergence results for ROI metric are shown in Fig. 5. The proposed ROAM achieves the best offline ROI metric and converges after 1000 iterations. Its ROI is much higher than OAM, indicating the importance of the ROI constraints. AUAF can converge fast after 100 iterations and achieves the second highest ROI. ROI of SHALE continues to increase with the iterations but is still far from ROAM after 1000 iterations. The offline ROI of ODBC is lower than ROAM, AUAF and SHALE. And HWM achieves the worst offline ROI.

**Fig. 6:**
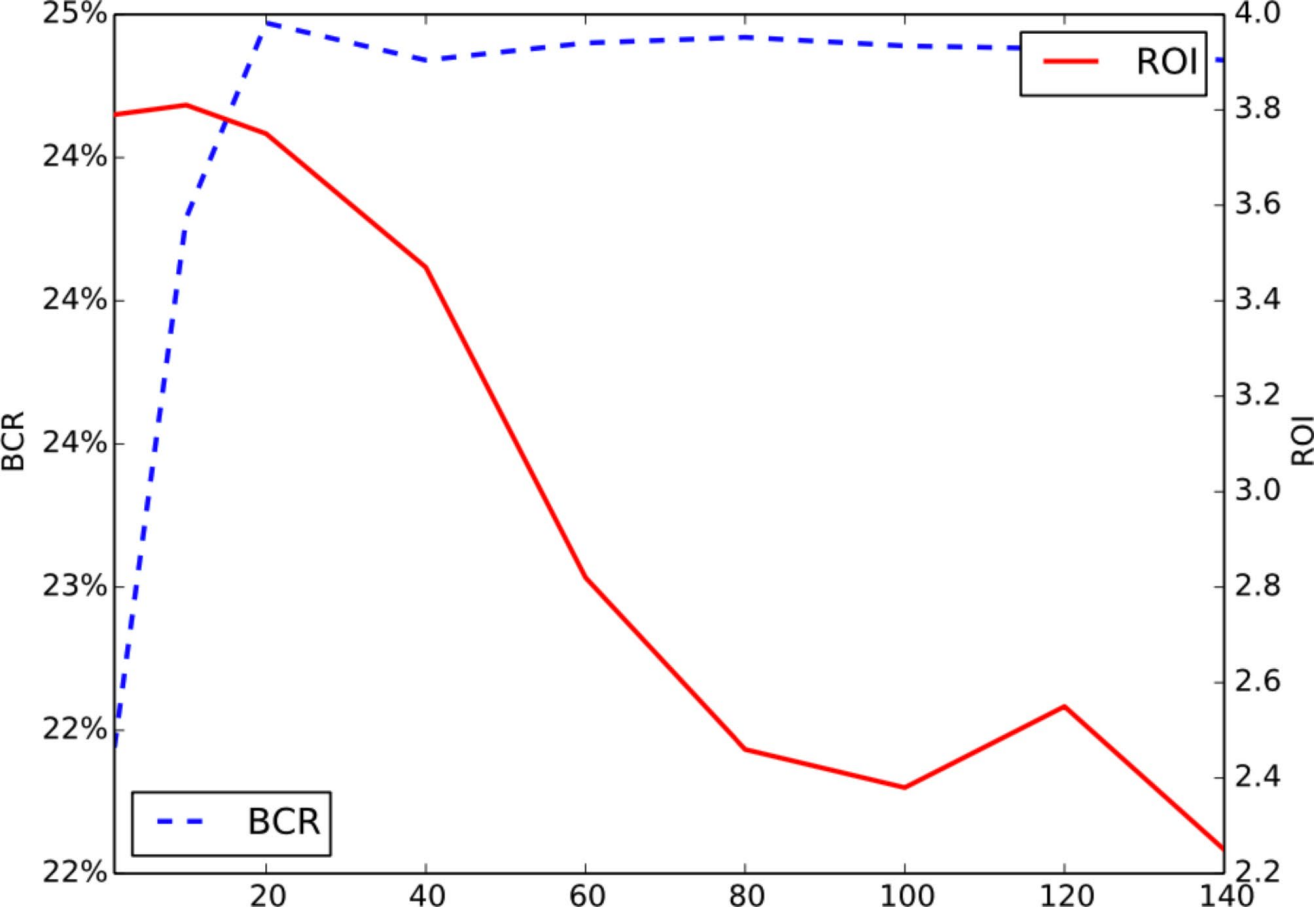
Sensitivity of Hyper-Parameter .

**Hyper-Parameter Sensitivity Analysis.** To conduct hyper-parameter sensitivity, we generate a synthetic dataset based on the dataset in^31^. According to our problem definition, larger lambda puts more weight on the revenue and ads will be displayed more times, disrupting user experience. As a result, CVR decreases with more ads shown, so does ROI since $\:ROI=\frac{price*CVR}{bid}$. Hence, is capable to balance ROI and revenue (or BCR). We evaluate the influence of hyper-parameter on ROAM in Fig. 6. The experimental optimal hyper-parameter = 20 is obtained by grid search.

**Time Consumption Analysis.** With millions of supply nodes and thousands of ad campaigns, our model takes less than 2 h to train for 1000 epochs with 1 server node (CPU) and 100 worker nodes (CPU). During the online serving stage, the serving time is negligible. The computation time is not a bottleneck.

To summarize, ODBC achieves the best revenue related metrics but with average performance on ROI. Best ROI is achieved by ROAM, followed by AUAF. AUAF is better than SHALE and HWM in terms of budget consumption rate and ROI. We will focus on the online performance of ODBC, ROAM and AUAF in the next section.

### Online A/B testing

We conduct the online experiments to compare the proposed ROAM with AUAF, ODBC, and a baseline approach, which is a probabilistic throttling method that maximizes the conversion rate from visit to purchase (i.e., pCTR, pCVR) based on the consumption rate of the budget. Note that for a fair comparison, each comparison method gets an equal share of the budget of each ad campaign and trains their allocation model using their respective auction logs. If all the methods share the same pool of budget, methods that consume budget faster would grab budget from methods that consume budget slower, making the comparison unfair.

**Fig. 7:**
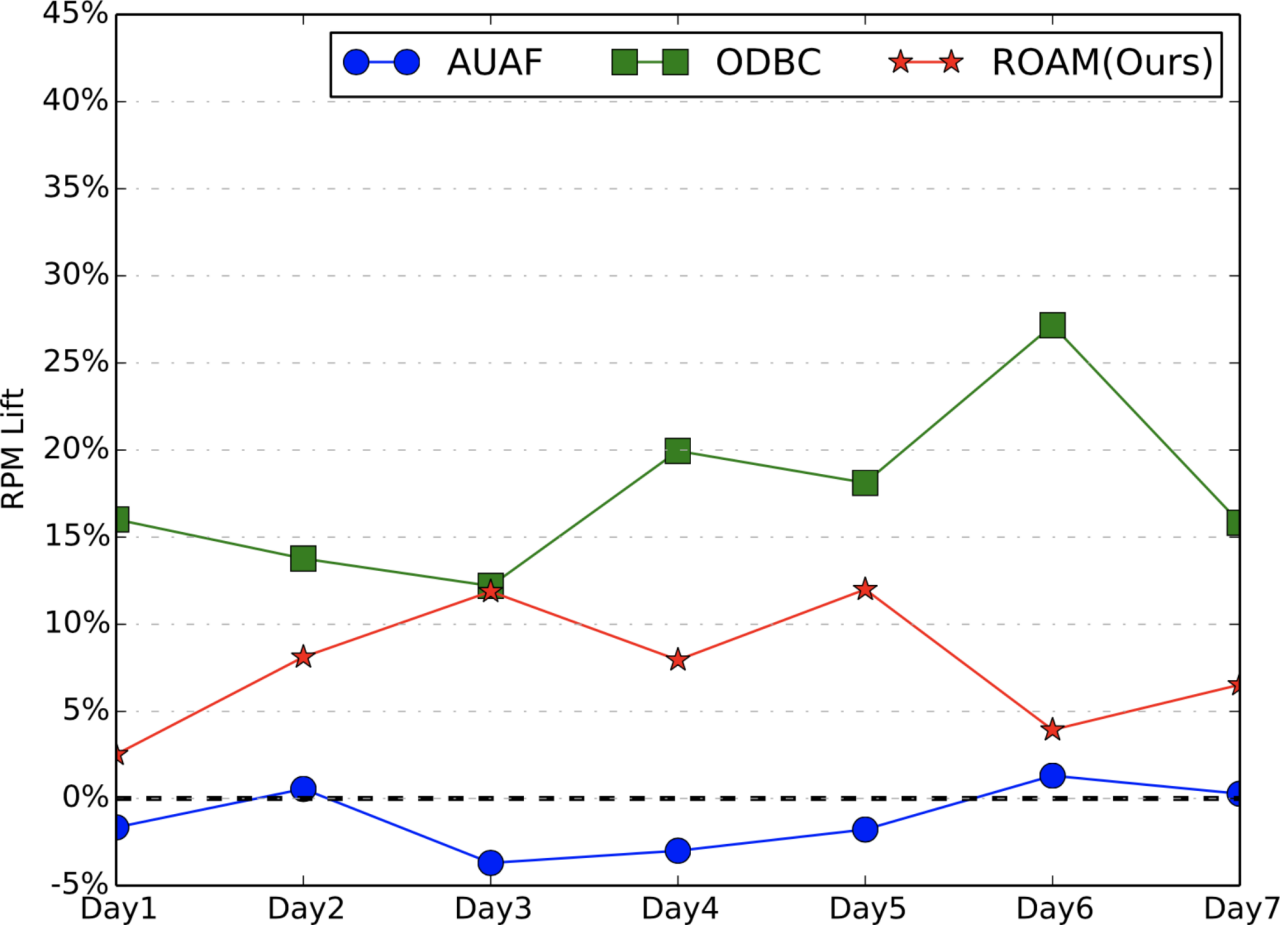
RPM lift of online A/B testing.

**Fig. 8:**
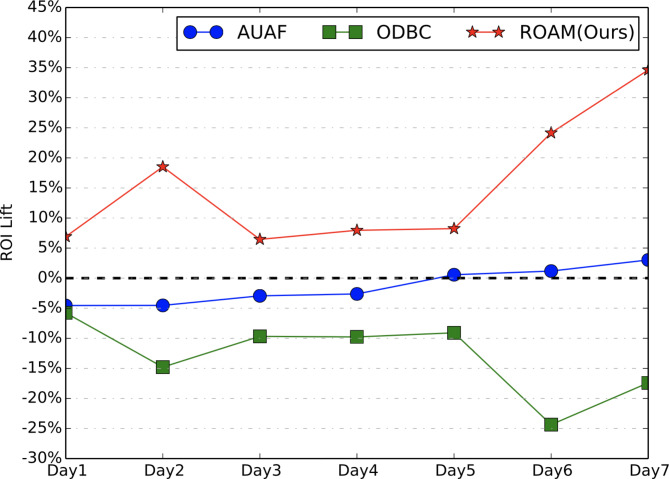
ROI lift of online A/B testing.

In online experiment, we focus on two metrics, i.e., RPM (with larger value indicating less user disturbance and higher effectiveness) and ROI. We conduct the online A/B testing for more than 7 days. Figures 7 and 8 show the results. Comparing to the probabilistic throttling-based baseline method, our proposed ROAM achieves stable and significant improvement on both on RPM and ROI. **Especially**,** ROAM is the only method that achieves a positive lift on RPM without sacrificing ROI.** Though ODBC has the highest RPM lift, it sacrifices a lot on ROI which is not acceptable. AUAF achieves no lift on both RPM and ROI.

It is worth mentioning that our model ROAM has been running stably in production for over three months since it was first launched.

## Conclusion

In this paper, we propose a new allocation model for sponsored search. It consists of two parts. For the offline part, an offline optimal solution is obtained by solving a constrained optimization problem from historical data with a quadratic objective and some linear constraints. It considers both platform revenue and user search experience in its goal, and makes a good trade-off between them by setting appropriate hyperparameter. An iterative algorithm is developed to efficiently solve this optimization problem in large scale. Instead of applying the offline solution directly online, we have designed some online strategies to address the potential conflict between the offline solution and GSP auction mechanism. Both offline and online experimental results show that our new model has made significant improvements on both platform revenue and advertiser ROI.

## Data Availability

The datasets used and/or analysed during the current study available from the corresponding author on reasonable request.
